# Microfluidic-Based Formulation of Essential Oils-Loaded Chitosan Coated PLGA Particles Enhances Their Bioavailability and Nematocidal Activity

**DOI:** 10.3390/pharmaceutics14102030

**Published:** 2022-09-23

**Authors:** Mohamed A. Helal, Ahmed M. Abdel-Gawad, Omnia M. Kandil, Marwa M. E. Khalifa, Alison A. Morrison, David J. Bartley, Gareth W. V. Cave, Hany M. Elsheikha

**Affiliations:** 1 School of Science and Technology, Nottingham Trent University, Nottingham NG11 8NS, UK; 2 Department of Parasitology and Animal Diseases, Veterinary Research Institute, National Research Centre, Giza 12622, Egypt; 3 Parasitology Department, Faculty of Veterinary Medicine, Cairo University, Giza 12211, Egypt; 4 Disease Control, Moredun Research Institute, Pentlands Science Park, Bush Loan, Penicuik, Edinburgh EH26 0PZ, UK; 5 Faculty of Medicine and Health Sciences, School of Veterinary Medicine and Science, University of Nottingham, Leicestershire LE12 5RD, UK

**Keywords:** essential oil, gastrointestinal nematodes, anthelmintic, PLGA, chitosan, microfluidic

## Abstract

In this study, poly (lactic-co-glycolic) acid (PLGA) particles were synthesized and coated with chitosan. Three essential oil (EO) components (eugenol, linalool, and geraniol) were entrapped inside these PLGA particles by using the continuous flow-focusing microfluidic method and a partially water-miscible solvent mixture (dichloromethane: acetone mixture (1:10)). Encapsulation of EO components in PLGA particles was confirmed by Fourier transform infrared spectroscopy, thermogravimetric analysis, and X-ray diffraction, with encapsulation efficiencies 95.14%, 79.68%, and 71.34% and loading capacities 8.88%, 8.38%, and 5.65% in particles entrapped with eugenol, linalool, and geraniol, respectively. The EO components’ dissociation from the loaded particles exhibited an initial burst release in the first 8 h followed by a sustained release phase at significantly slower rates from the coated particles, extending beyond 5 days. The EO components encapsulated in chitosan coated particles up to 5 μg/mL were not cytotoxic to bovine gut cell line (FFKD-1-R) and had no adverse effect on cell growth and membrane integrity compared with free EO components or uncoated particles. Chitosan coated PLGA particles loaded with combined EO components (10 µg/mL) significantly inhibited the motility of the larval stage of *Haemonchus contortus* and *Trichostrongylus axei* by 76.9%, and completely inhibited the motility of adult worms (*p* < 0.05). This nematocidal effect was accompanied by considerable cuticular damage in the treated worms, reflecting a synergistic effect of the combined EO components and an additive effect of chitosan. These results show that encapsulation of EO components, with a potent anthelmintic activity, in chitosan coated PLGA particles improve the bioavailability and efficacy of EO components against ovine gastrointestinal nematodes.

## 1. Introduction

Parasitic gastroenteritis is a major challenge to small ruminant production and has been associated with substantial economic losses and welfare concerns [1]. The abomasal hematophagous nematode *Haemonchus contortus* (*H. contortus*) is the most economically important parasite, responsible for a tremendous reduction in sheep productivity [2]. Anthelmintics are routinely used to control parasitism but concerns over residues, and development of multidrug-resistant parasite strains threaten the sustainability of this approach, driving the need for an alternative and more sustainable parasite control measures [3]. Researchers have been delving into the possibility of using bioactive phyto-chemicals as an alternative to anthelmintics, hoping to reduce the reliance on synthetic chemicals [4].

Essential oils (EOs) and their constituents possess remarkable biological activities [5,6,7]. In a previous study, the essential oil coriander and three EO components (linalool, geraniol and eugenol) were found to have a nematocidal effect against several ovine gastrointestinal nematodes [8]. However, the use of EOs is hampered by the limited thermal and chemical stability, poor efficacy at low concentrations, and the narrow margin of safety [9]. Encapsulation of EOs within a polymeric matrix can protect the bioactive compounds of EOs, enhance their biological properties, reduce toxicity, and facilitate a targeted and controlled delivery [10]. Among the common biodegradable polymers, poly(lactic-co-glycolic acid) (PLGA) is widely used as a polymeric carrier in hydrophobic drug delivery, such as EO components, for production of micro- or nanoparticles [11].

PLGA particles can be formulated by conventional methods, such as emulsification-solvent evaporation and nanoprecipitation techniques. However, these methods are not reproducible, and associated with broad size distribution and less entrapment efficiency [12]. In contrast, microfluidic technology enables the production of monodisperse particles with varying sizes and morphological features in high-throughput and reproducible batches via controlling the fluid flow [13]. Two microfluidic methods are often used to produce either micron-scale particles with a higher encapsulation efficiency and drug loading capacity (droplet-based flow focusing method) or nano-scale particles with a relatively low entrapment efficiency and drug loading capacity (continuous flow-focusing method) [14]. Synthesis of particles with a specific size without compromising their entrapment and loading capacities can be achieved by a continuous flow-focusing method, using a partially water-miscible solvent mixture as the dispersed phase, which produces a consistent flow when mixing with a continuous aqueous phase, producing discrete microdroplets that lead to the formation of nano-scale particles [15].

The post-ruminal (abomasal) delivery of drugs via the oral route is often hampered by mechanical disruption by rumination, chemical degradation, microbial fermentation, and ruminal retention [16]. Coating the surface of PLGA particles with chitosan can transform anionic PLGA particles into cationic, chitosan (CS)-coated PLGA particles (CS-PLGA) [17]. This modification improves the mucoadhesive properties of the particles, prolongs the retention time within the mucosal tissue, and sustains the release kinetics [18]. Additionally, chitosan has a good solubility in acidic media [19], enabling the release of EO components in the abomasum, where *H. contortus* live. Chitosan can also enhance the penetration of the entrapped drug to the submucosa via opening the tight-junctions of the intestinal barrier [20].

In the present study, we tested the hypothesis that encapsulating EO components (linalool, eugenol, and geraniol) into CS-PLGA particles improves EO component bioavailability and maintains their efficacy against gastrointestinal nematodes, particularly *H. contortus*. We produced micro and nano-particles of CS-PLGA encapsulating linalool, eugenol, and geraniol, individually or combined. The hydrodynamic size, polydispersity index (PDI), and ζ-potential of the CS-PLGA particles were characterized. We also investigated the entrapment efficiency, loading capacity, and in vitro release kinetic of EO components from loaded particles using gas chromatography mass spectrometry (GC-MS) and UV- vis spectrophotometer. A further solid-state characterization was performed using Fourier transform infrared (FT-IR) spectroscopy, thermogravimetric analysis (TGA), and X-ray powder diffraction (XRD). The antiparasitic activity of the particles was tested against larval and adult stages of *H. contortus* and the structural changes of the cuticle was examined using scanning electron microscopy (SEM). Finally, we assessed the effect of particles loaded with EO components on host cell viability, proliferation, membrane integrity, drug permeability, and cellular uptake using a bovine gut cell line (FFKD-1-R).

## 2. Materials and Methods

### 2.1. Chemicals and Reagents

The following chemicals were purchased from Sigma-Aldrich (St. Louis, MO, USA): Poly(D,L-lactide-co-glycolide) (PLGA; lactide:glycolide 65:35, MW 40–75 KDa), Poly(vinyl alcohol) (PVA; 87%–89% hydrolyzed, MW = 13–23 KDa), sucrose, sodium tripolyphosphate (TPP; MW = 367.86), medium molecular weight chitosan (MW = 50–190 KDa), polysorbate 80, span 20, fluorescein 5(6)-isothiocyanate (FITC), clove oil (*Eugenia caryophyllata*), lavender oil (*Lavandula angustifolia*), pure components (~100% purity), and analytical standards of linalool and geraniol. Pure eugenol was obtained from flourochem Ltd. (Glossop, UK). The essential oils citronella (*Cymbopogon nardus*) and coriander (*Coriandrum sativum*) were purchased from Oils4life Ltd. (Great Yarmouth, UK). Dichloromethane (DCM) and acetone were obtained from Fisher Scientific Inc. The analytical standards of eugenol were purchased from the European Pharmacopoeia (EP) reference standards.

For cell culture work, bovine intestinal cell line of jejunum/ileum (FKD-1-R; Cat No. CCLV-RIE 0971) was kindly provided by Friedrich-Loeffler-Institute, Federal Research Institute for Animal Health, Germany. Dulbecco’s Modified Eagle’s Medium—low glucose (DMEM), heat-inactivated fetal bovine serum (FBS), trypsin-EDTA Solution 10X, Dulbecco’s phosphate buffer saline (DPBS), trypan blue, and 3-(4,5-dimethyl-2-thiazolyl)-2,5-diphenyl-2H-tetrazolium bromide (MTT) solution were purchased from Sigma-Aldrich. Penicillin-streptomycin (10,000 U/mL) and dimethyl sulfoxide (DMSO—cell culture grade) were purchased from Fisher Scientific—Gibco™. IncuCyte^®^ Cytotox red dye was purchased from Sartorius, USA. Phalloidin-iFluor 555 reagent (ab176756) and DAPI (ab285390) were obtained from Abcam, Cambridge, UK.

### 2.2. Microfluidic System and Chip

**Figure 1:**
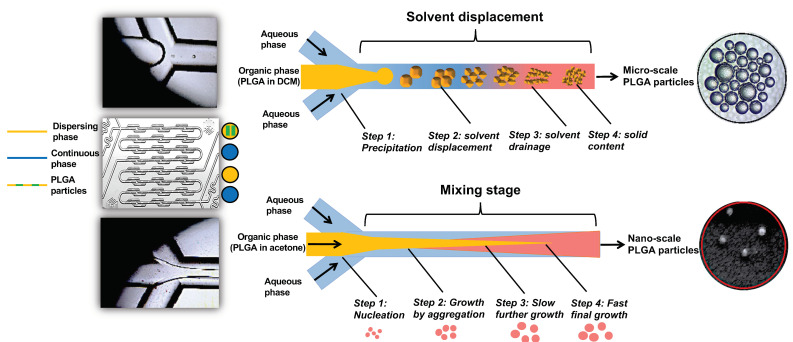
A schematic diagram of the microfluidic chip used (hydrophilic micromixer) and the process of the formulation of micro and nanoparticles.

The micromixer system, developed by The Dolomite Centre Ltd., Royston, UK, was used to prepare PLGA particles at micro and nanoscales. The system uses a microfluidic hydrodynamic focusing (MHF) technique and consists of a micromixer chip (Dolomite, 3200401), a hydrophilic compact glass microfluidic device that enables rapid mixing of two fluids with 12 herring bone mixing stages to produce the particles in a controlled and reproducible way. The chip was assembled with the H Interface (Dolomite, Part No. 3000155) and two Linear Connectors 4-way (Dolomite, Part No. 3000024). The fluids were delivered by two pressure pumps (Fluika) through FEP Tubing, 1/16 × 0.25 mm and the stream was controlled by 2-way in-line valves and visualized via a high-speed digital microscope. The process of particle formulation using a micromixer chip by solvent displacement (acetone) or solvent diffusion (DCM) is illustrated in Figure 1.

### 2.3. Chemical Analysis

The chemical composition of EOs citronella, clove, coriander, and lavender was determined by qualitative and quantitative methods. The headspace (Turbo-Matrix HS 16 sampler, PerkinElmer, Waltham, MA, USA) coupled with gas chromatography-mass spectrometry (Clarus^®^ SQ 8 GC/MS PerkinElmer, USA) was used to identify the volatile components of each EO [21]. The quantitative analysis of EOs was carried out on gas chromatography (Agilent 6890N GC system) equipped with a flame ionization detector (FID) and HP-5MS capillary column (30 m × 0.25 mm i.d., 0.25 µm film thickness) as described previously [22] with minor modifications. Briefly, 1 µL of 1% EO diluted in methanol was injected in splitless mood. The injector temperature was set at 250 °C. Helium was used as the carrier gas flowing at 1 mL/min and pressure of 7.6 bar. The initial temperature of the column was 50 °C for 2 min and increased to 200 °C at a rate of 5 °C/min and maintained for 3 min at the final temperature. The electron impact mass spectra were scanned at 70 ev in the mass range of 40–600 units. Each sample was analyzed in triplicate with a total run time of 37 min. The chemical components were detected via matching their mass spectra and retention times to the NIST library data. The peak area of each EO component was compared to a calibration curve constructed from the values of five known concentrations of a reference standard of the respective EO component.

### 2.4. Preparation of PLGA and CS-PLGA Particles

To synthesize PLGA particles, PLGA 1 and 2% (*w*/*v*) was dissolved in organic solvent (organic phase) and rapidly mixed with water (aqueous phase) in the Dolomite micromixer chip to formulate the oil-in-water (O/W) emulsion. The organic solvents used were dichloromethane (DCM) and acetone either individually or in combinations (1:10 and 1:20, respectively) to optimize the formulation condition. Due to the hydrophobic nature of the EOs, the EO components (geraniol, linalool, and eugenol) were dispersed in the organic phase with different concentrations (0.25, 0.5, and 1%). The working concentrations were determined based on the particle size and entrapment efficiency percentage (EE%) of the EO components chosen for chitosan coating.

The particles were prepared via microfluidic hydrodynamic focusing as previously described [14], with some modifications. Based on the preliminary results of optimization, 100 mg PLGA was dissolved in 10 mL solvent (DCM: acetone mixture; 1:10) to form the organic phase. The aqueous phase was prepared by dissolution PVA 1% (*w*/*v*) in deionized water. The two fluids were mixed in the dolomite’s micromixer system at a flow rate 1000 µL/min with an operating pressure 500:250 mbar for dispersing to a continuous phase. For encapsulation, the EO components were mixed individually or in combination with the organic phase 0.5% (*v*/*v*). The hydrophilic lipophilic balance (HLB) was adjusted by addition of span 80 to the organic phase (1% *v*/*v*) and tween 80 to the aqueous phase (1% *v*/*v*) to help emulsification of oils in distilled water. To modify the PLGA particles surface, chitosan was dissolved in 1% glacial acetic acid (0.25%), stirred overnight, and mixed with the aqueous phase. The PLGA particles were collected from the chip’s output to a preloaded reservoir with 10 mL of 0.1% PVA solution. For cross-linking of chitosan, 0.4% of TPP solution was gradually added to the emulsion with continuous stirring to form ionic gelation. The organic solvent was then evaporated overnight under a moderate magnetic stirring and the particles were recovered by centrifugation at 10,000 rpm for 30 min at 4 °C and washed three times with distilled water. The pellet was suspended in distilled water to form a homogenous suspension and subsequently lyophilized in a freeze dryer at −65 °C for 72 h (Labconco, Eaton Socon, UK).

### 2.5. Characterization of the Prepared Particles

#### 2.5.1. Photon Correlation Spectroscopy and Zeta-Potential Analysis

The size, polydispersity index (PDI), distribution, and homogeneity of the prepared particles were examined using dynamic light scattering (DLS) and photon correlation spectroscopy (PCS), while the Zeta (ζ)-potential was determined using a Zetasizer Nanoseries ZS (Malvern instruments, Malvern, UK). Freshly prepared and lyophilized particles were diluted with distilled water to a final concentration of 1 mg/mL to obtain evenly distributed particles. The measurement was performed at room temperature with a refractive index of 1.33 and a viscosity of 0.8872 cp. Each sample was analyzed in triplicate and the results are presented as the means ± standard deviations (S.D.).

#### 2.5.2. Entrapment Efficiency (EE%) and Drug Loading Capacity (LC%)

The encapsulation efficiency and EO components content of the prepared particles were determined by GC/MS chromatography as described previously [7], with some modifications. Briefly, 5 mg of the lyophilized sample was dissolved in 1 mL methanol to degrade the polymer and release the EO components. This step was enhanced by subsequent sonication–vortexing cycles. To precipitate the polymer, the samples were centrifuged at 13,000 rpm for 15 min and the concentration of EO components was quantified in the clear supernatant using a calibration curve constructed by using five known concentrations of a reference standard. The plain particles were treated using the same procedure and used as a blank. Triplicate of each prepared particle were analyzed. The EE% and LC% were calculated according to the following formulas [20]:

EE (%) = (Loaded amount of EO components)/(Initial amount of EO components) × 100

LC (%) = (Total amount of loaded EO components)/(Mass of the lyophilized particle) × 100

#### 2.5.3. Morphology of the Particles

The surface morphology and actual sizes of uncoated PLGA and CS-PLGA particles were examined using Scanning Electron Microscopy (SEM) (JEOL JSM-7100F, Tokyo, Japan). Fine particles of each sample were mounted on EM metal stubs previously covered with double-sided carbon tapes and coated with a thin gold layer using sputter coater (Q150R ES, Quorum, Madrid, Spain). The particles were photographed at an acceleration voltage of 5 KV.

#### 2.5.4. Fourier Transform Infrared (FTIR) Spectroscopy

The chemical composition of pure EO components, polymers, the used chemicals, and the prepared particles were determined by using FTIR spectroscopy (Spectrum™ 3, Perkin Elmer, USA) at a resolution of 4 cm^−1^ and wavenumber range of 400–4000 cm^−1^.

#### 2.5.5. Thermogravimetric Analysis (TGA)

The thermal stability of pure EO components, polymers, the used chemicals, and the prepared particles were detected by using Thermogravimetric analyzer (TGA 4000, PerkinElmer, USA). Approximately 10 mg of the lyophilized powder/10 μL of liquid chemicals were placed in the platinum crucible and heated up gradually at an increasing rate of 10 °C/min from 30 to 600 °C under helium atmosphere to detect the weight loss %, which reflects the encapsulation efficiency of EO components. 

#### 2.5.6. Powder X-ray Diffraction (XRD) Analysis

The solid-state patterns and the degree of crystallinity for the used chemicals and the prepared particles were determined by using X-ray diffractor (Rigaku SmartLab SE, Tokyo, Japan) with operating conditions 9 mA, 45 kV and Cu Kα radiation (λ = 1.5 Å). The finely powdered chemicals or particles were scanned at an angle of 2θ and over 5° to 50° with a speed of 0.03°/s.

#### 2.5.7. Localization of EO Components and Chitosan in PLGA Particles

To localize the chitosan and EO components distributions within the PLGA particles, chitosan was conjugated with a fluorescent dye (FITC) based on the reactivity of the FITC thiocyanate group to primary amino group of chitosan as described previously [23,24]. The FITC labeling was performed using a molar ratio of 1:100 (FITC: monomer unit). The FITC solution was prepared by dissolving 1.7 mg FITC in 6.8 mL of dehydrated methanol, then 3.865 mL was added dropwise to the chitosan solution (40 mg chitosan in 0.1 M acetic acid solution) with constant stirring at 300 rpm/4 h in the dark at room temperature. The FITC-chitosan was precipitated by 0.5 M NaOH (pH 10), centrifuged at 10,000 rpm /10 min three times, and dialyzed against distilled water for 3 days using 3–5 kDa pore size dialysis tubing. The labeling and purification efficiency was evaluated in the recovered polymer and dialysis water using fluor-spectrometer (NanoDrop™ 3300, Thermo Scientific, Oxford, UK) at λexc 495 nm and λemi 519 nm, respectively. The FITC labelled chitosan was then used to coat the PLGA particles loaded with EO components following the same abovementioned procedures. The synthetic fluorescent labelled particles were photographed using EVOS FL microscope (Life Technologies, Carlsbad, CA, USA) and the fluorescence profile was generated using ImageJ (Software 1.48 V).

### 2.6. In Vitro Release Analysis

The cumulative release profile of the encapsulated EO components from triplicate samples of PLGA and CS-PLGA particles was detected in 5 mg dried particles/2 mL of three different buffer solutions (phosphate buffer saline, pH 7.2; acetate buffer, pH 5.5; and hydrochloric acid-potassium chloride buffer, pH 2.0) and incubated up to 120 h in a thermomixer (Eppendorf, Hamburg, Germany) at 37 °C with stirring for 300 rpm. The alkaline and acidic buffers were chosen to mimic the ruminal and abomasal pH, respectively with stirring to mimic the gut movement. At predetermined time points (0, 0.5, 1, 2, 4, 8, 24, 48, 72, 96, and 120 h) each sample was centrifuged at 10,000 rpm for 10 min and 1 mL of the buffer was collected and replaced with the same volume of a fresh buffer. The released EO components were measured using a UV–vis spectrophotometer (NanoDrop™ One, Thermo Scientific, UK) at wave lengths 210, 227, and 282 nm for geraniol, linalool, and eugenol, respectively. At the end of the experiment, the remaining pellets of particles were dried and treated with methanol to quantify the retained EO components content in the particles using GC/MS. The cumulative release percentage at each time point was calculated based on the ratio of EO components released to the initial encapsulated amount.

### 2.7. Cellular Studies

The cytocompatibility of the prepared particles was evaluated via treatment of bovine intestinal cell culture with free EO components, drug-free (plain) particles and loaded particles at different concentrations to evaluate the cytotoxicity, cell proliferation, cell membrane integrity, and cellular uptake.

#### 2.7.1. Culturing and Maintenance of FKD-1-R Cells

The bovine gut cell line (FFKD-1-R) was maintained in DMEM (low glucose), supplemented with 10% heat inactivated FBS and 1% penicillin-streptomycin. Cultures were seeded at 10^3^ cells/cm^2^ and incubated at 37 °C in 5% CO_2_ humidified incubator. Subculturing (passaging) was performed when the culture reached 90% confluency after 56–72 h at a splitting ratio of 1:2. The confluent cell monolayers were carefully aspirated from their culture medium, rinsed twice with DPBS and treated with 2–3 mL of pre-warmed trypsin-EDTA solution at 37 °C until the cells appear rounded and detached under an inverted microscope. The effect of trypsin was quenched by adding 6–8 mL complete DMEM medium and the cell suspension was centrifuged at 1,000 rpm for 3 min. The cell pellet was resuspended in growth medium, and the viable cells were counted by mixing 10 µL of cell suspension with equal volume of trypan blue (0.4%) and counting was carried out using TC20 Automated Cell Counter (Bio-Rad Laboratories, Hercules, CA, USA). The cells were either cryopreserved (2 × 10^6^ cells/mL complete culture medium with 7.5% DMSO) or plated till the logarithmic growth phase for use in the experiments.

#### 2.7.2. Optimization of Cell Seeding Density

The seeding density of FKD-1-R cells was optimized by seeding the cells at six different densities: 0.5, 1, 2, 3, 4, 5 and 6 × 10^4^ cells/100 µL/well into 96-well tissue culture microtiter plates. The plates were incubated at 37 °C in a 5% CO_2_ humidified incubator. The degree of confluency and cell viability was examined by using the MTT assay at 24, 48, and 72 h.

#### 2.7.3. Cell Viability

The cytotoxic effects of free EO components, loaded (PLGA and CS-PLGA) particles against FKD-1-R was assessed by MTT reduction assay as described previously [25]. Briefly, FKD-1-R cells were seeded at a density of 2 × 10^4^ cells/100 µL/well into 96-well tissue culture microtiter plates and the cells were incubated for at least 16 h undisturbed at 37 °C under a humidified atmosphere of 5% CO_2_. The free EO components and prepared particles were UV-sterilized and serially diluted with culture medium to 1.25, 2.5, 5, and 10 μg/mL. The cell culture growth medium was carefully aspirated, then replaced with the test medium (100 µL/well) containing various concentrations of EO components in triplicate and kept for 6, 24, and 48 h at the same conditions. Loaded particles were compared with plain particles as a negative control and the blank wells contained only DMEM. After the indicated exposure time, 20 µL MTT solution (5 mg/mL in PBS) was added to the foil-wrapped plate with a further incubation for 2 h followed by gentle aspiration of the media and the purple formazan crystals formed at the bottom of the wells were solubilized by adding 100 μL DMSO/well with plate shaking at a moderate speed for 10 min. The optical density was measured using a plate reader (Clariostar, Cologne, Germany) at a wavelength of 570 nm. The absorbance values were corrected for background absorbance from the blank wells containing medium only and correlated to the absorbance of negative control wells. All experiments were performed three separate times each in triplicate.

#### 2.7.4. Live Cell Imaging (IncuCyte) for Proliferation Analysis and Cell Membrane Integrity

A real-time live cell analysis was used to evaluate the effect of exposure of FKD-1-R cells to different concentrations of PLGA and CS-PLGA particles loaded with EO components compared with cells treated with the same concentrations of free combined EO components. The analysis was based on the procedures described previously [26]. Firstly, the FKD-1-R cells were seeded into 96-well tissue culture microtiter plates at a seeding density 1 × 10^4^ cells/100 µL/well and incubated for at least 16 h to allow cell adherence. The medium was carefully aspirated and replaced with fresh medium supplemented with IncuCyte^®^ Cytotox Red (250 nM concentration for counting dead cells) and containing the combined EO components at concentrations 1.25, 2.5, 5, and 10 μg/mL either in the loaded particles or in free form (triplicate well for each condition). The plain particles were used as negative control and the blank wells contained only DMEM. The plates were incubated in IncuCyte^®^ Live-Cell Analysis System (Essen BioScience, Berlin, Germany) fitted within CO_2_ incubator set at 37 °C and 5% CO_2_. The integrated IncuCyte^®^ S3 software photographed three fields of view of each well every 3 h with 10x objective lens over 48 h. The levels of confluency and growth curves were generated for evaluation of cell proliferation % compared to negative controls. The cytotoxic effect of the loaded particles was estimated as red fluorescence area (µm^2^), which reflects the loss of cell membrane integrity and uptake of the nucleic acid dye, yielding a hundred-fold increase in the fluorescent signal.

#### 2.7.5. Cellular Localization of CS-PLGA Particles

Cellular tracking of FITC-labelled CS-PLGA particles loaded with EO components was visualized using confocal laser scanning microscopy (CLSM) as described previously [27]. The FKD-1-R cells were seeded into a special cell imaging 96-well tissue culture plates (Black wall/clear flat bottomed; Falcon^®^353219, Corning, NY, USA) at density of 10^3^ cells/200 µL/well and incubated at 37 °C in 95% humidity and 5% CO_2_. The following day, the cell culture medium was carefully aspirated and replaced with fresh prewarmed medium containing FITC-labelled CS-PLGA particles in concentrations of 0, 1.25, 2.5, and 10 µg/mL. After 24 h incubation, the media were gently removed, and the cells were washed three times in DPBS followed by fixation with 4% methanol-free formaldehyde in DPBS at room temperature for 30 min. The fixed cells were washed several times before permeabilization with 0.1% Triton X-100 in DPBS for 5 min. The permeabilized cells were rinsed with DPBS 3 times/each 5 min followed by staining of the actin filaments (F-actin) using phalloidin and the nuclear counterstain DAPI. Firstly, a 100 µL of diluted phalloidin (1 µL of 1000x phalloidin mixed in 1 mL PBS + 1% BSA) was added to each well and the cells were incubated at room temperature for 90 min. Finally, the cells were washed once with DPBS and treated with DAPI (1:1000 in PBS) for 10 min at room temperature in a dark place followed by several washes in DPBS prior to cell imaging using a laser scanning confocal microscope (Leica SP5, Wetzlar, Germany), fitted with appropriate filter for each fluorescence signals at Ex/Em = 350/470, 495/519 and 556/574 nm for DAPI, FITC and phalloidin, respectively.

### 2.8. Parasitological Studies

#### 2.8.1. Source of the Parasites

Third larval stage of *H. contortus* were kindly provided by Moredun Research Institute in Scotland. The adult abomasal nematodes (*H. contortus* and *T. axei*) were collected from a local abattoir (Najib & Sons Ltd., Derby, UK). The larvae and adult nematodes were washed with saline, double distilled water, and then centrifuged at 700× *g* for 5 min, followed by three washes with PBS containing 4% penicillin and streptomycin. Only actively motile adults and larvae were used in the study.

#### 2.8.2. Larval and Adult Worm Motility Assay

The larval and adult worm motility assay was performed as described previously [28]. Briefly, larvae or adult worms were placed in 48 multi-well tissue culture plates at a density of 3 worms or 50 larvae per well and exposed to free or encapsulated EO components at different concentrations (1.25, 2.5, 5 and 10 μg/mL diluted in 2% PBS-Tween 80 + 4% penicillin/streptomycin). The diluent was used as negative control and the positive control wells included parasites treated with 20 mg/mL levamisole in the same diluent. The plates were incubated at 37 °C in 5% CO_2_ incubator for 24 h and the motility was observed under a stereomicroscope (Leica Microsystems, Buckinghamshire, UK) and was ascertained by absence of mobility over a period of 5–6 s. The number of motile and immotile worms were counted for each concentration upon prodding and observation for 5 s to calculate the immobility index (%) = number of immobile worms/total number of worms × 100. This experiment was performed three independent times, each in three technical replicates. The higher the motility inhibition %, the better the anthelmintic activity.

#### 2.8.3. Scanning Electron Microscopy (SEM) 

The larvae and adult worms treated with encapsulated EO components were examined using SEM as described previously [29]. Briefly, samples were washed three times with distilled water and fixed in 3% glutaraldehyde in 0.1 M cacodylate buffer (CACO; pH 7.4) for 24 h followed by washing twice with CACO buffer prior to post fixation in 1% osmium tetroxide. Samples were washed three times with distilled water and dehydrated in a graded series of ethanol. The samples were then infiltrated with hexamethyldisilazane (HMDS) (Acros, Renningen, Germany) for 5 min twice. The samples were mounted on metal stubs adherent to the surface of carbon tapes for coating with a 10 nm layer of gold in a sputter coater (Q150R ES, Quorum, Madrid, Spain). The morphological features of the parasite cuticle were photographed using a scanning electron microscope (JEOL JSM-7100F, Japan) at an accelerating voltage of 20 kV.

### 2.9. Statistical Analysis

All statistical analyses were performed using GraphPad Prism 9 Software. Unless otherwise stated, data were presented as means ± standard errors (SE) of at least 3 independent experiments. One- or Two-Way ANOVA and multiple comparison followed by the Dunnett test were used to determine significant differences between means (*p* < 0.05). Sigmoidal inhibition dose–response curves were calculated using a variable slope nonlinear regression model. Four-parameter logistic equation was applied using global curve-fitting, with the bottom of the curves constrained to zero. For each treatment, the half maximal inhibitory concentration (IC_50_) and R^2^ values were calculated.

## 3. Results

### 3.1. Compositional Analysis of Crude EOs

The chromatograms of crude EOs revealed 17–18 components from citronella, clove, and coriander, while 48 components were identified in lavender. The GC/MS analysis indicated that linalool was the most abundant compound in the coriander and lavender EOs, representing 37.25% (5.3 mg/mL) and 32.48% (3.6 mg/mL), respectively. The major compounds in citronella were citronellal and geraniol, which represented 37.26% (4.76 mg/mL) and 17.22% (2.2 mg/mL) of the total oil, respectively. Eugenol represented 52% of the clove (8.83 mg/mL). A typical gas chromatogram of the crude EOs together with the retention times for the major components is shown in Figure S1. The three EO components (geraniol, linalool and eugenol) represented in Figure S2 were quantified in the crude EOs, working solutions and encapsulated particles based on standard calibration curves (Figure S3).

### 3.2. Optimization of the Conditions for Particle Formulation

The optimal conditions to produce the particles were determined via the particle size and PDI as shown in Table S1. The following is the recommended conditions for the organic phase (1% polymer in DCM: acetone mixture (1:10), 1% span 80 and 0.5% EO components) and the aqueous phase (PVA 1% in deionized water and 1% tween 80). The optimization of microfluidic condition was determined at a flow rate of 1000 µL/min with pressure 500:250 mbar for dispersing to continuous phase.

### 3.3. Characterization of the Prepared Particles

**Figure 2:**
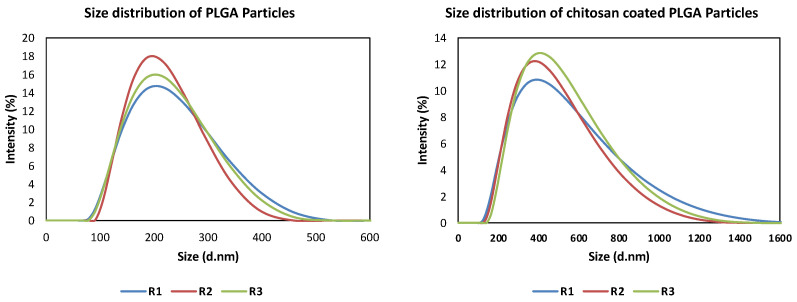
Size distribution by intensity of the uncoated PLGA and CS-PLGA particles. R1, R2, and R3 denote the replicates R1, R2, and R3, respectively.

The colloidal properties of the prepared particles resulted in particles with average sizes of 273 nm and PDI of 0.15 in case of uncoated PLGA particles, whereas the CS-PLGA particles had a mean size of 335 nm and PDI of 0.19 (Figure 2). The analysis also showed that the particles were produced in nanoscales (~97%) and microscales (~3%) based on size distribution by numbers. The surface charge of PLGA particles was shifted from the negative (−23.3 ± 5.01) to the positive side of zeta potential (24.7 ± 9.06) after surface modification by chitosan.

### 3.4. Entrapment Efficiency and Drug Loading Capacity

**Figure 3:**
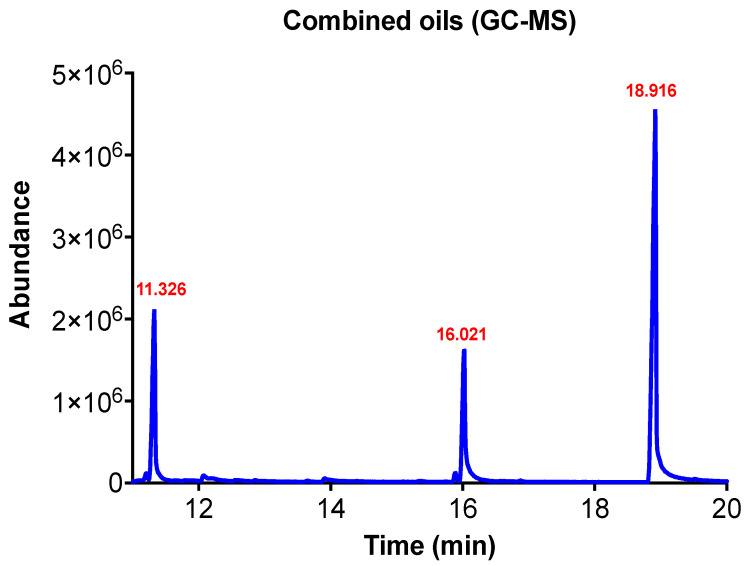
Chromatogram of the combined EO components encapsulated in the prepared particles with retention times 11.326, 16.021, and 18.916 for linalool, geraniol, and eugenol, respectively.

GC/MS analysis was used to determine the amount of EO components entrapped within the particles by using a standard calibration curve and a regression line equation with R^2^ of 0.998 (Figure S4). The results showed that the particles had encapsulation efficiencies 95.14, 79.68 and 71.34% for eugenol, linalool, and geraniol, respectively. The loading capacities were 8.88%, 8.38%, and 5.65% in particles entrapped with eugenol, linalool, and geraniol, respectively. The chromatogram of the encapsulated EO components is presented in Figure 3 and the results indicate that the maximum EE% and LE% were identified at 0.5% EO components.

### 3.5. Ultrastructural Chanracteristics of the Particles

**Figure 4:**
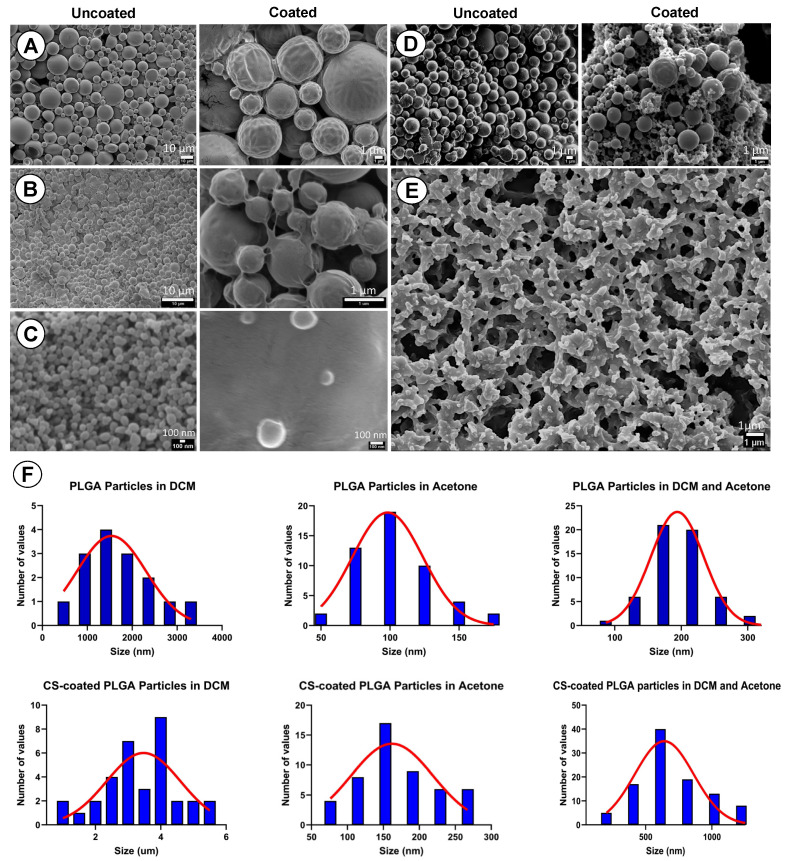
SEM micrographs showing the PLGA particles prepared using different conditions. (**A**) Uncoated and CS-PLGA particles prepared by conventional method. (**B**) Uncoated and CS-PLGA particles prepared by microfluidics using DCM as EO solvent. (**C**) Uncoated and CS-PLGA particles prepared by microfluidics using acetone as EO solvent. (**D**) Uncoated and CS-PLGA particles prepared by microfluidics using a DCM: acetone (1:10) as EO solvent. (**E**) Scaffold structure of chitosan. (**F**) Histogram of the actual particle size distribution in the EM images analyzed using ImageJ software.

The SEM results showed that uncoated and CS-PLGA particles are produced at nanometric and micrometric scales based on the solvent used in particles’ preparation. The particles appeared spherical, with a relatively smooth surface and narrower size distribution when prepared using the microfluidic method compared with the conventional method (Figure 4). The freeze-dried chitosan showed a network structure scaffold which formed a film surrounding the PLGA particles (Figure 4F). The actual mean diameter of the particles was obtained by analyzing SEM micrographs using ImageJ software, with nanoscale particles (200 nm) and particles with a larger size (650 nm) for those prepared by mixed solvents (Figure 4D).

### 3.6. FTIR Spectroscopy

**Figure 5:**
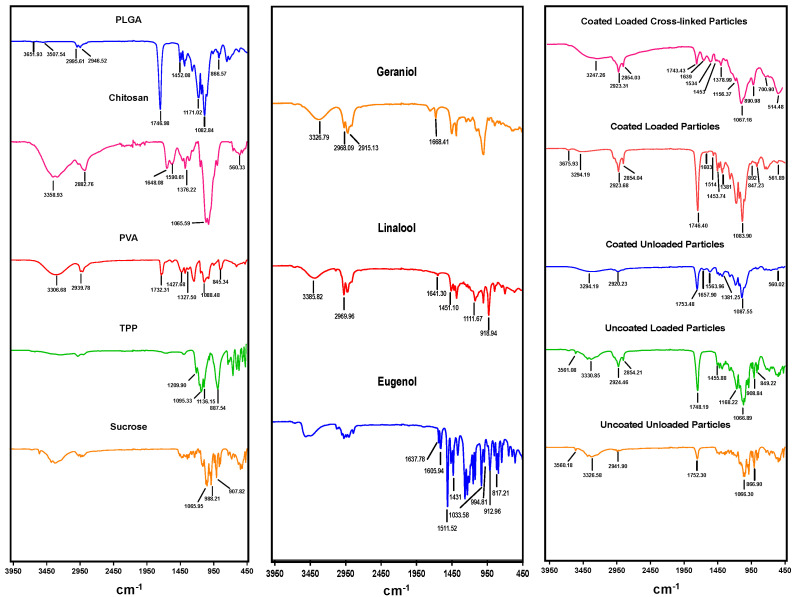
Fourier transform-infrared spectra of pure chemicals used to prepare the particles, EO components, plain and loaded PLGA particles (uncoated or chitosan coated), and crosslinked particles prepared in the study.

The FTIR spectra of pure PLGA, PVA, chitosan, and EO components were analyzed to confirm the encapsulation of EO components within the prepared particles, successful coating of PLGA particles with chitosan, and cross-linking with TPP (Figure 5). The characteristic peaks of PVA were detected at 3306 cm^−1^, 1732 cm^−1^, 1427 cm^−1^, and 1327 cm^−1^ corresponding to stretching vibration of –OH group, stretching vibration of –C = O group, and bending vibrations of –CH2 and –CH3 groups, respectively. The chitosan exhibited two sharp peaks at 1648 cm^−1^ and 1590 cm^−1^ relating to the primary and secondary amides, and signals of C–H stretch and C–H bend at wavenumbers 2882 cm^−1^ and 1376 cm^−1^, respectively, as well as characteristic peak of N–H stretch at 3358 cm^−1^. The PLGA was recognized with absorption bands at 1746 cm^−1^ for C = O stretching, 866–1452 cm^−1^ for C-H bending, 3507 cm^−1^ for OH stretching, and 2946 cm^−1^ for CH2 stretching. The absorption bands of 1209 cm^−1^, 1136 cm^−1^, 1095 cm^−1^ and 887 cm^−1^ were observed in the TPP spectra corresponding to P = O, -PO2, -PO3 and P-O-P stretching, respectively. Sucrose was used as a cryoprotectant and showed specific bands at 907 cm^−1^, 988 cm^−1^ and 1065 cm^−1^ in the fingerprint region. The three EO components showed a broad peak at 3300 cm^−1^ relating to alcohol OH stretch. Linalool and geraniol spectra indicated intense peaks in the region of 918 cm^−1^, 1111–1451 cm^−1^ and 2969 cm^−1^ due to stretching vibrations of C-C, C-O, and C-H groups, respectively. Eugenol had spectral signal frequencies at 817 cm^−1^, 994 cm^−1^, 1431 cm^−1^, and 1605 cm^−1^ assigned for ring deformation, trans CH out-of-plane, CH2 vibration, and presence of C = C group region, respectively. The spectrum of CS-PLGA particles compared with uncoated and crosslinked particles confirmed chitosan adsorption to the surface of particles and successful TPP crosslinking. On the other hand, the spectra of loaded particles compared with the corresponding plain particles showed the encapsulation of EO components.

### 3.7. Thermal Analysis

**Figure 6:**
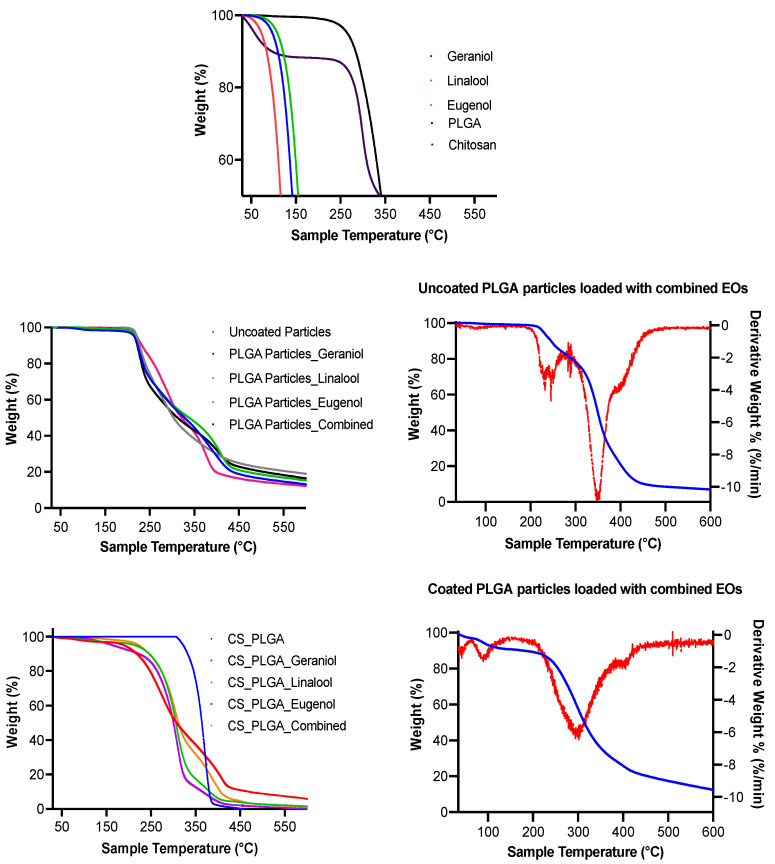
Thermogram of chemicals used to prepare the particles, EO components, plain and loaded PLGA particles (uncoated or coated), and the corresponding derivatives; thermogravimetry (DTG) analysis on the right side.

As shown in Figure 6, TGA analysis revealed the thermal stability of pure EO components, prepared particles, and the chemical derivatives used in the formulations based on the degradation temperatures where the significant weight loss was recorded. Derivative thermogram indicated one mass loss step with peak degradation temperatures between 108–162 °C, 78–135 °C, 103–178 °C, and 198–398 °C for geraniol, linalool, eugenol, and PLGA, respectively. Regarding the TGA curve of chitosan, two endothermic peaks were identified at 90 °C and 299.7 °C. On the other hand, PLGA or CS-PLGA particles loaded with EO components had a characteristic peak of EO components within the endothermic peak of PLGA or chitosan indicating the encapsulation of EO components, confirming the previous results of loading capacity 21%. The degradation temperature of the encapsulated EO components was shifted toward a higher temperature than free EO components (210 °C in PLGA particles and 250 °C in CS-PLGA particles), indicating enhancement of thermal stability of EO components.

### 3.8. Powder XRD Analysis

**Figure 7:**
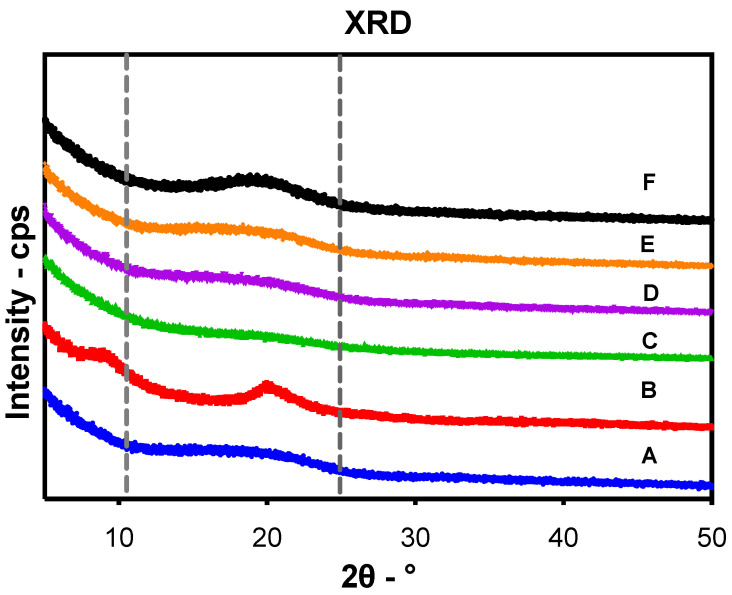
Powder X-ray diffractograms of (**A**) PLGA, (**B**) chitosan, (**C**) uncoated PLGA particles, (**D**) uncoated PLGA particles loaded with combined oils, (**E**) CS-PLGA particles, and (**F**) CS-PLGA particles loaded with combined EO components.

The XRD patterns of PLGA, chitosan, uncoated and CS-PLGA particles, either plain or in powder form, are illustrated in Figure 7. Because of the amorphous structure of PLGA and chitosan, no characteristic diffraction peaks were recorded in the XRD pattern. The PLGA exhibited one diffraction peak at 20° which was also observed in chitosan along with another peak at 10° on the 2 Theta scale. The loaded PLGA particles (uncoated or coated) showed a broader peak at 20° compared with those of the plain particles, indicating the encapsulation of EO components within the particles. 

### 3.9. Localization of EO Components and Chitosan in PLGA Particles

**Figure 8:**
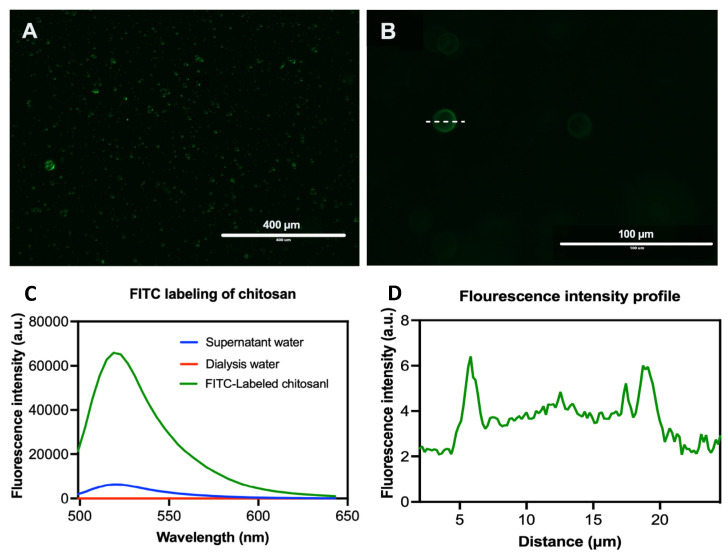
Representative fluorescence images of FITC-labeled CS-PLGA particles (**A**,**B**). FITC labelling evaluation (**C**) and fluorescence intensity profile across the center of the particles (**D**).

The fluorometric analysis showed successful labeling of chitosan with FITC before being applied in the coating of the plain or loaded PLGA particles (Figure 8A). The chitosan formed a thin uniform layer surrounding the PLGA particles loaded with EO components as shown by the fluorescence intensity profile across the center of the particles (Figure 8B). The purification efficiency of the labelled chitosan was monitored by measuring the fluorescence intensity of FITC (excitation 499 and emission 519) in the supernatant after centrifugation (maximum intensity = 6300), dialysis water (zero intensity) and the rescued polymer (maximum intensity = 66,000) (Figure 8C). The fluorescent dye was concentrated at the surface of the particles while the particle’s core was free or emitted a faint green fluorescence of FITC (Figure 8D).

### 3.10. In Vitro Release Profile of Encapsulated EO Components

**Figure 9:**
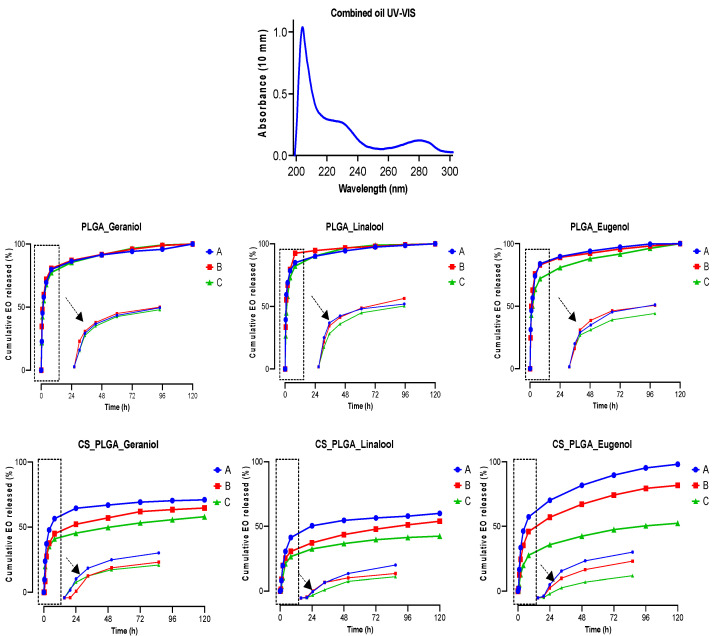
In vitro release kinetics of EO components from the uncoated and CS-PLGA particles over 120 h in hydrochloric acid-potassium chloride buffer, acetate buffer, and phosphate buffer saline denoted as A, B and C, respectively. The data are presented as average cumulative EO components release % with standard errors (1.4:2.8%). The top graph shows the absorbance band of geraniol, linalool, and eugenol detected at wavelengths of 210, 228, and 282 nm, respectively.

The release profiles of the encapsulated EO components from the uncoated and CS-PLGA particles were calculated based on a standard calibration curve of EO components using UV-vis spectrophotometer (Figure S4). As shown in Figure 9, both types of particles exhibited a biphasic release pattern with an initial burst release phase in the first 8 h followed by a sustained release phase till the end of experiment (120 h). Generally, eugenol was released faster than geraniol and linalool. In the rapid release phase, the three EO components were released up to 82.8 ± 2.7%, 85.4 ± 6.2%, and 77.2 ± 5.1% in PLGA particles, which significantly decreased to 51.7 ± 9.0, 40.5 ± 8.6, and 31.7 ± 8.1 in CS-PLGA particles when the particles were in vitro released in hydrochloric acid-potassium chloride, acetate and phosphate buffers, respectively. In the second release phase, the three EO components were completely released from the PLGA particles in 120 h regardless of the releasing buffer, while the cumulative release percentages of the EO components in the CS-PLGA particles after 120 h were 76.3 ± 9.6%, 66.8 ± 4.0%, and 50.9 ± 7.9% in buffers with pH 2.0, 5.5 and 7.2, respectively.

### 3.11. Cytotoxicity Assay

**Figure 10:**
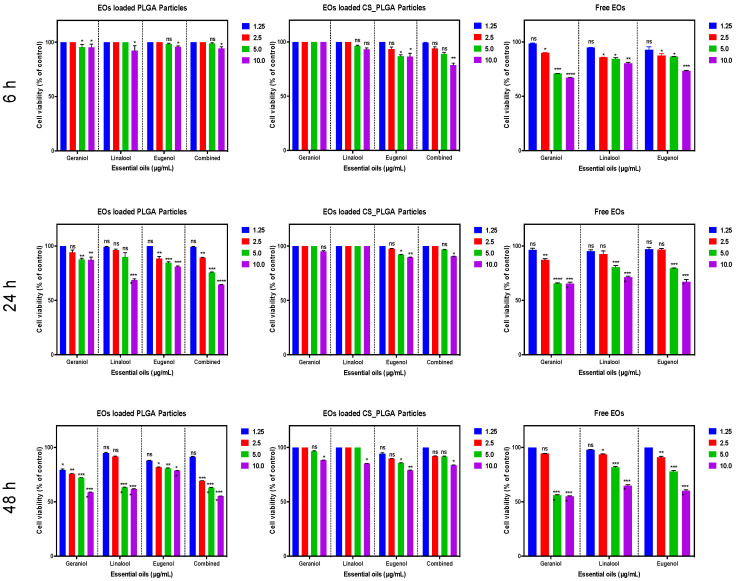
Cytotoxic effect of free and encapsulated EO components (within uncoated and CS-PLGA particles) on FKD-1-R cells measured by MTT assay. Results are expressed as the means of cell viability (% of control) ± standard errors. The level of difference was measured by one-way ANOVA followed by Dunnett’s multiple comparisons. Asterisks denote statistical significance (* *p* < 0.05; ** *p* < 0.01; *** *p* <0.001; **** *p* < 0.0001 compared to control groups and ns denotes non-significant).

Based on the results of the optimization experiment, a seeding density of 2 × 10^4^ cells/well was chosen because it was the minimum number of cells that allows the detection of differences in cell growth at 48 and 74 h (Figure S5). The cytotoxic effect of free and encapsulated EO components (at concentrations 1.25, 2.5, 5.0, and 10.0 μg/mL) in uncoated and CS-PLGA particles on the viability of FKD-1-R cells was estimated by using MTT assay at 6, 24, and 48 h post exposure (Figure 10). The results showed that the free essential oils exerted a significant cytotoxicity at a concentration > 2.5 μg/mL over the entire period of the experiment. The encapsulation of EO components within the PLGA induced a modest decrease in cell viability after treatment for 6 h at a concentration of 10 μg/mL and the maximum cytotoxic effect was observed at 24 and 48 h even at <2.5 μg/mL concentration of EO components, probably attributed to the rapid burst release phase of the PLGA particles (77.2–85.4% of EO components were released). Interestingly, the encapsulated EO components within CS-PLGA particles did not show any significant difference in cell viability percentage compared to the negative control up to 5 μg/mL at the three-time points, which may be attributed to the delayed onset and reduced intensity of the burst release phase, in addition to the slower release of EO components from the CS-PLGA. 

### 3.12. Live Cell Imaging Analysis

**Figure 11:**
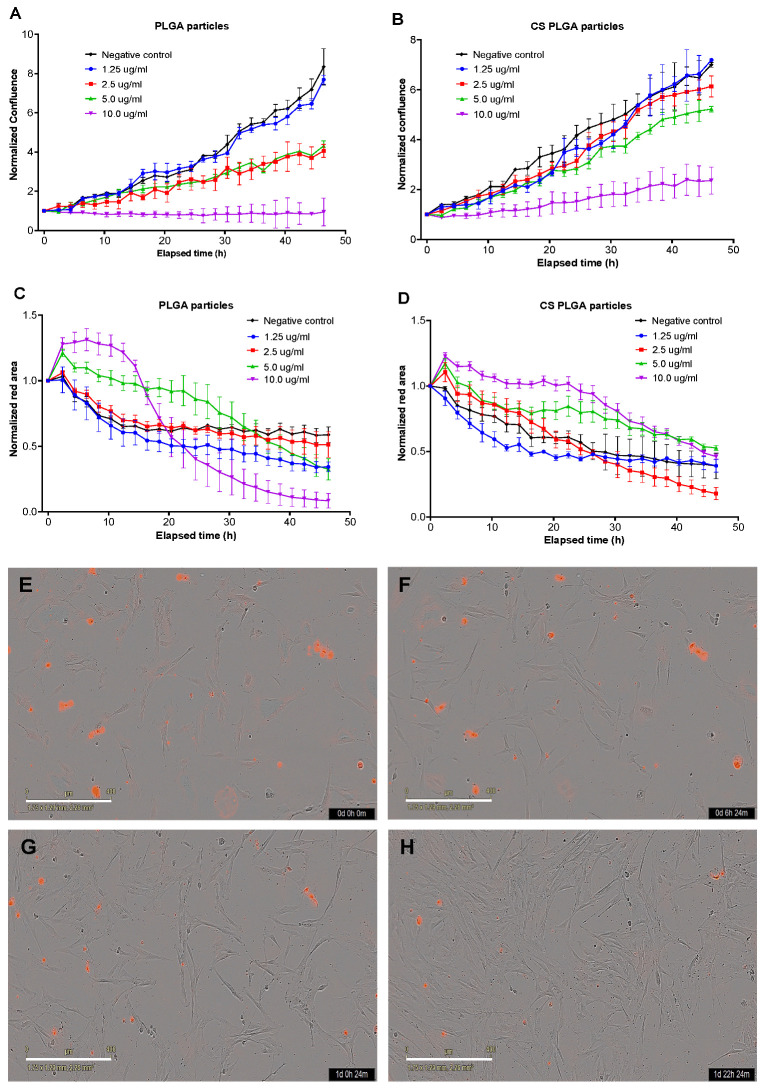
Live cell imaging (IncuCyte) analysis showing cellular proliferation (**A**,**B**) and cytotoxicity (**C**,**D**). The results show the effect of treatment of bovine intestinal FKD-1-R cells with uncoated or CS-PLGA particles encapsulating the indicated EO components measured at 3 h intervals for 48 h. The normalized results of the mean cell confluence or values of red areas ± standard errors were plotted in curves produced by the integrated IncuCyte software against the elapsed time. (**E**–**H**) Representative images (10x objective) show the degree of proliferation and red fluorescent areas in wells treated with 1.25 µg/mL for 0, 6, 24 and 48 h, respectively. Scale bars = 400 µm.

The proliferation and cytotoxicity levels after 48 h treatment of FKD-1-R cells with uncoated or CS-PLGA particles loaded with different concentrations of combined EO components (1.25, 2.5, 5, and 10 µg/mL) were measured using real-time live cell imaging (Incucyte), which is based on measuring the degree of confluency in phase contrast and the cell death (red areas) in the red fluorescence (Figure 11). The untreated FKD-1-R cells grew in a dose-dependent manner whereas the growth rate of treated cells decreased proportional to increasing the concentrations of EO components. In case of the PLGA particles, cell proliferation was inhibited after 30 h of incubation with combined EO components (2.5 and 5 µg/mL), while 10 µg/mL caused a complete suppression of cell growth at 0 h. On the other hand, no significant differences were observed in the level of confluency of cells treated with CS-PLGA particles loaded with up to 5 µg/mL of EO components after 48 h. The cell growth patterns in either uncoated or CS-PLGA particles loaded with 1.25 µg/mL of EO components were similar to that of the negative control (Figure 11A,B). Cytotoxicity assessment by MTT assay showed that the number of dead cells was initially increased during the first 8 h of incubation of cells treated with uncoated or CS-PLGA particles encapsulated with 5 or 10 µg/mL of EO components. In contrast, live cell imaging revealed no significant cytotoxic effect (*p* < 0.05) for both particles loaded with EO components at <5 µg/mL compared to the negative control (Figure 11C,D). The analysis of cell viability was based on the quantification of total red area per well at different treatment concentrations at a 3 h interval.

### 3.13. Cellular Localization of CS-PLGA Particles

**Figure 12:**
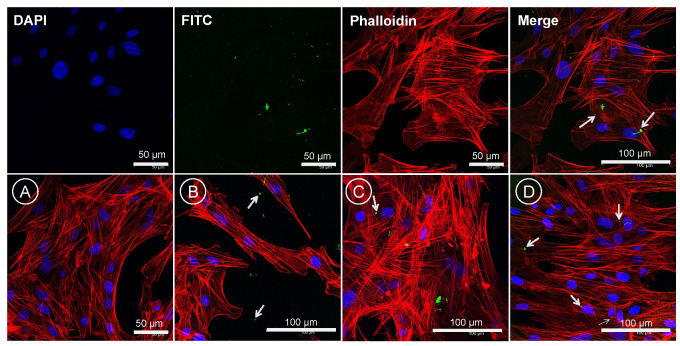
Confocal Laser Scanning microscope (CLSM) images of cellular localization of CS-PLGA particles. The images in the top row show 24 h-treated FKD-1-R cells with FITC-labelled CS-PLGA particles (green), cytoplasmic actin filaments (red) and nuclear counterstain (blue). The images in the bottom row represent the confocal images of (**A**) negative control (particles-free wells), (**B**) particles localized intercellularly, (**C**) adsorption of the particles on cell membrane, and (**D**) perinuclear localization of the particles. The green fluorescence of FITC-labeled particles is indicated by arrows.

A confocal laser scanning microscope was used to analyze the fixed FKD-1-R cells stained with DAPI (blue fluorescence of nuclei), phalloidin (red fluorescence of cytoplasmic actin filaments), and FITC (green fluorescence of labelled chitosan in CS-PLGA particles). Despite the washing and processing of cells, the particles were retained and detected as green, fluorescent spots or aggregates (Figure 12). The CS-PLGA particles were adsorbed onto the surface of FKD-1-R cells. The confocal images showed micro particulates were intercellularly localized, while the nano particles were taken up by the FKD-1-R cells and appeared around the cell nucleus.

### 3.14. Larval and Adult Worm Motility

**Figure 13:**
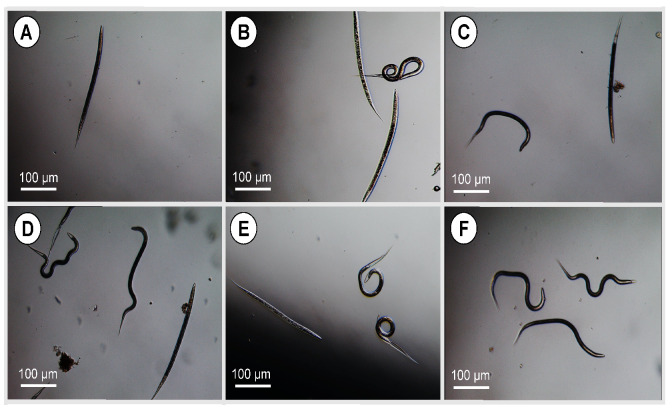
Microscopical examination of third stage larvae (L3s) of *H. contortus* after exposure to CS-PLGA particles loaded with combined EO components at (**A**) 10 µg/mL, (**B**) 5 µg/mL, (**C**) 2.5 µg/mL, and (**D**) 1.25 µg/mL. (**E**) L3s treated with 20 mg/mL levamisole were used as a positive control. (**F**) Untreated L3s were used as a negative control. Dead L3s in the treated and positive control samples were immobile upon prodding. Negative control larvae were actively motile even after several days of incubation. Scale bar = 100 µm.

**Figure 14:**
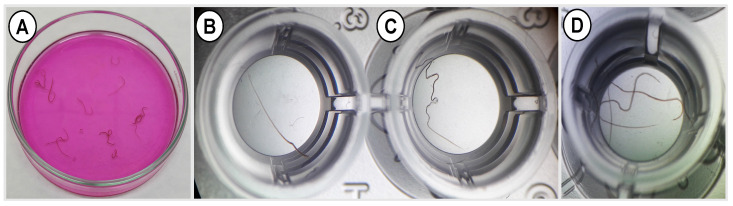
Adult worm motility assay showing worms treated with CS-PLGA particles encapsulating combined EO components. (**A**) *H. contortus* worms exposed to 10 µg/mL. 4x magnification. (**B**–**D**) *T. axei* worms exposed to 10, 5, and 2.5 µg/mL, respectively. 40x magnification.

The free and encapsulated EO components (individually or combined) showed a significant (*p* < 0.05) inhibitory activity on larval motility of *H. contortus* (Figure 13). The PLGA particles loaded with geraniol, linalool, eugenol individually, or in combination (1.25 µg/mL) inhibited the larval motility by 30.8%, 42.9%, 39.1%, and 46.7%, respectively. Interestingly, coating the PLGA particles with chitosan has slightly increased the efficacy of the encapsulated EO components, which exhibited immobility index of 44.4%, 46.7%, 43.9%, and 47.6%, respectively, at the same concentration (i.e.,1.25 µg/mL). At a higher concentration, 10 µg/mL, the larval motility was inhibited by 75% and 76.9% in loaded PLGA and CS-PLGA particles with the three oils, respectively. The free EO components had a strong effect on larval motility, reaching 96.1% inhibition, compared with the encapsulated EO components. The IC_50_ values were relatively higher in the loaded PLGA particles than in the CS-PLGA particles and free EO components (Table S2). We observed a synergistic effect of combining EO components and an additive effect of chitosan as was evident by the reduced IC_50_ values compared with free individual EO components and uncoated PLGA particles. Regarding the adult worm motility assay, the particles loaded with combined EO components completely inhibited the worm motility 24 h post-exposure of adult *T. axei* and *H. contortus* to 5 and 10 µg/mL (Figure 14).

### 3.15. Scanning Electron Microscopy

**Figure 15:**
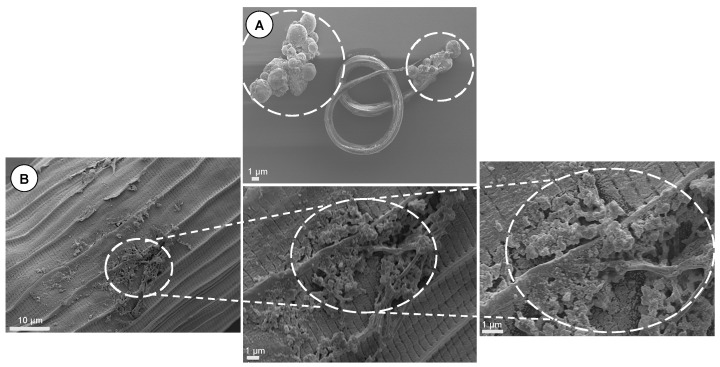
SEM micrographs of parasites treated with CS-PLGA particles loaded with combined EO components. The particles are deposited on the cuticular surface of (**A**) larva and (**B**) adult worm.

**Figure 16:**
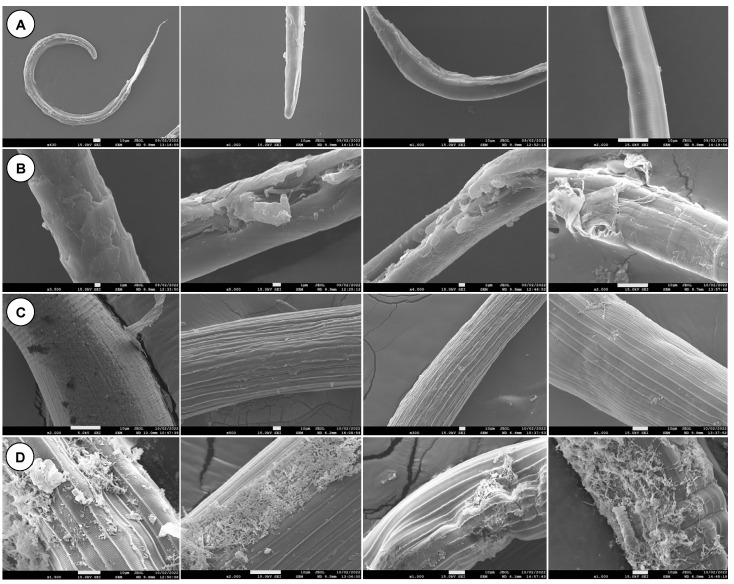
SEM micrographs of parasite stages treated with CS-PLGA particles loaded with combined EO components. (**A**) Negative control larvae (Scale bar = 10 µm). (**B**) Treated larvae (Scale bar = 1 µm). (**C**) Negative control adult worms (Scale bar = 10 µm). (**D**) Treated adult worms (Scale bar = 10 µm).

SEM micrographs of parasites treated with CS-PLGA particles loaded with combined EO components are shown (Figure 15 and Figure 16). Both micro and nanoparticles were firmly adsorbed to the cuticle of larvae and adult worms and were not affected by the subsequent washing procedures during sample preparation. The untreated larvae or adult worms exhibited no morphological or structural changes. The parasite stages exposed to particles loaded with EO components exhibited significant cuticular damage, loss of cross striations in the treated larvae, and wrinkled cuticles in treated adults. The larval stages displayed more severe and extensive damage in the muscular layer compared with adult worms. All body parts of both parasite stages (i.e., anterior end, posterior end, and middle part) were equally affected by the loaded particles. 

## 4. Discussion

The global sheep industry faces a considerable challenge in regard to the control of gastrointestinal nematodes particularly *H. contortus* [2]. EOs and their components have been previously proposed as a potential source of alternative antiparasitic agents against gastrointestinal nematodes [30]; however, more comprehensive studies are still required to evaluate their chemical composition, anthelmintic activities, and toxicological effects. The crude EOs of coriander, citronella, clove, and lavender are composed of a complex mixture of oxygenated and hydrocarbon compounds, with a few components constituting 20–70% of the content and dictating the biological activity of EOs, such as linalool, geraniol, and eugenol, which have shown anthelmintic activity [31]. The compositional analysis of EOs can vary with extraction method, harvesting stage and environmental factors [32]. Also, despite the effectiveness of EOs, their applications are limited due to poor water solubility, degradation, high volatility and considerable toxicity [33]. The present study used a micromixer chip and continuous flow-focusing microfluidic platform for encapsulation of three EO components within the polymeric carrier PLGA and examined the effect of encapsulation on the EO’s stability and bioavailability. This approach generated reproducible, stable, and uniform particles with high mono-dispersity (PDI < 0.2), in agreement with previous findings [34]. In the present study, a HLB was achieved for each EO component because it is a key factor for maintaining the equilibrium between the dispersing and continuous phases and the optimal HLB values were obtained by using the most efficient surfactants polysorbate 80 and span 20, as described previously [35].

The results of optimization study showed that the size of the produced particles is controllable based on the solvent used in the dispersing phase, the amount of loaded EO components, and PLGA concentrations. Overall, a higher polymer concentration formed larger particles. Likewise, an increase of the loaded EO components increases the particle size. The nature of the organic phase is the main factor for production of a tunable size range related to the water miscibility of the organic solvent [36]. When DCM alone was used as an organic solvent, the production of microparticles was obtained. In contrast, smaller particles were obtained with water-miscible solvent such as acetone which may be attributed to a faster diffusion in aqueous phase and nucleation of nanoparticles [37]. We thus used a partially water-miscible solvent mixture of DCM and acetone which creates an interfacial tension between the dispersing and continuous phase, while mixing in a microfluidic chip with entrapping more EO components. This has led to the synthesis of submicronic PLGA particles achieving high encapsulation efficiency up to 95.14% and loading capacity as high as 22.91%, in agreement with a previous study [14].

Surface modification of PLGA particles by chitosan can improve their mucoadhesiveness and increases the residence time of the particles within the mucosal tissue, achieving a slow, sustained release of the EO components, which ultimately improve the particles’ effect [20]. Remarkably, coating of PLGA particles with chitosan resulted in a significant increase in the hydrodynamic diameters of the particles with a positive zeta potential, suggesting the physical adsorption of chitosan layer to the surface of the particles [25]. The surface charge of PLGA particles was shifted from negative (−23.3 ± 5.01) to positive zeta potential (24.7 ± 9.06), which is attributed to the functionalization of PLGA free carboxyl groups by the amine group of chitosan [38]. This in turn facilitates electrostatic binding of particles to negatively charged mucin and cell membranes, thus enhancing the retention of particles within gut mucosa [39]. Morphologically, the uncoated and CS-PLGA particles had a uniform spherical shape with smooth surfaces, which minimizes tissue irritation compared with uneven or crystal-shaped particles [40]. In case of CS-PLGA particles, the chitosan coat formed an evenly distributed layer surrounding the dense core of the PLGA and EO components as shown by SEM analysis. This pattern was confirmed by FITC labelling of chitosan, which showed green fluorescence concentrated at the surface of the particles while the particle’s core was free or emitted a faint green fluorescence of FITC, in agreement with others [24]. 

FTIR spectroscopy analysis revealed the characteristic peaks of EO components, PLGA, and chitosan, confirming the entrapment of the EO components within the loaded particles and successful coating of PLGA particles with chitosan, and verifying cross-linking with TPP. All prepared particles exhibited a strong peak at ~1746 cm^−1^ of the carboxylic group of PLGA, while surface-modified particles developed a broader band at 3294 cm^−1^ assigned to the amino (−NH2) group of chitosan and sharp peaks at 1648 cm^−1^ and 1590 cm^−1^ related to primary and secondary amides, in agreement with previous results [27]. Some of the peaks were shifted and other absorption bands were detected or have increased intensities in particles loaded with EO components, confirming the incorporation of EO components within the polymer matrix, such as stretching vibration of C-H, C-O, and CH2 groups in EO components at wavenumbers of 2854 cm^−1^, 1168–1455 cm^−1^, and 1431 cm^−1^, respectively [41]. On the other hand, spectral signal frequencies were considerably altered in cross-linked particles, including two peaks at 1534 cm^−1^ and 1156 cm^−1^, indicating the complex interaction between phosphoric groups of TPP and NH^3+^ groups of chitosan, suggesting the successful TPP crosslinking with chitosan in the prepared particles [42].

The encapsulation of EO components enhanced their thermal stability, whereas the degradation temperatures for free EO components were significantly increased up to 210 and 250 °C when entrapped in the uncoated and CS-PLGA particles, respectively and the percentage of weight loss that reflects the loading capacity of EO components was 22%, supporting the GC/MS data. These results corroborate previous findings [43], showing improvement of thermal stability of encapsulated EO components. Despite the amorphous structure of PLGA and chitosan, the broader diffraction peak at 20° in the XRD pattern of loaded particles compared to those of plain particles showed the encapsulation of EO components within the particles as previously reported [44]. Another advantage of encapsulation is the controlled release of EO components to obtain a sustained release and targeted delivery to achieve the desired therapeutic effect. In this regard, a biphasic release pattern of EO components from the uncoated and CS-PLGA particles was detected with an initial burst release phase in the first 8 h (due to rapid diffusion of the excess adsorbed oils on the particle’s surface) followed by a sustained release phase (due to slow diffusion of the encapsulated oils within the polymeric matrix core) [45]. Coating of PLGA particles with chitosan lowered the intensity of the burst effect and the overall rate of EO components release, in accordance with previous findings [25]. This effect was attributed to the formation of physical barrier by chitosan that hinders the diffusion of EO components from the particles into the release medium. Moreover, the release kinetics of EO components from CS-PLGA particles was pH-dependent with a significant rapid release in acidic than alkaline or neutral buffer in contrast to PLGA particles, where the release kinetic exhibited a similar pattern, regardless of the releasing buffer. These findings are possibly related to the good solubility of chitosan in acidic pH due to protonation of a primary amine group of chitosan at low pH and the formation of a complex insoluble network in alkaline or neutral media [46]. In addition, the hydrophilic nature and swelling behavior of PLGA particles influenced the release rate of EO components due to the permeability of water into the particle matrix with the initiation of polymer degradation and the liberation of EO components faster than the CS-PLGA particles [47].

Some EO components have some degree of toxicity, which limits their use as anthelmintics [48]. Therefore, the cytotoxic effects of free EO components, loaded PLGA, and CS-PLGA particles on adherent FKD-1-R were assessed at 6, 24, and 48 h using MTT assay. These incubation periods were chosen to allow a reasonable amount of the entrapped EO components to be released from the particles and to cover both rapid and slow phases of release [25]. The results showed that encapsulation of EO components not only improved the sustained release profile, but also minimized the potential toxic effect of EO components [49]. In addition, surface coating of PLGA particles with chitosan reduced the cytotoxicity of the entrapped EO components compared with uncoated PLGA particles, which may be attributed to the intensity onset of the burst-release phase along with slower release of EO components from the CS-PLGA particles compared with the uncoated ones [50]. These results were further confirmed by live cell imaging (IncuCyte) analysis to evaluate the effect of exposure of FKD-1-R cells to the particles loaded with EO components on cell proliferation and cell membrane integrity. The data showed that the inhibition of cell growth was recorded at 10 µg/mL with a significantly antiproliferative effect up to 2.5 µg/mL in uncoated PLGA particles and up to 5 µg/mL in CS-PLGA particles. This effect may be related to cell membrane lysis due to the reduction of the membrane’s surface tension [51]. This disruption of cell membrane integrity allows the IncuCyte^®^ Cytotox Red dye to penetrate the cells and intercalates with the nucleic acid, yielding a 100- to 1000-fold increase in fluorescence, which in turn can be quantified and used as to assess changes in cell viability [52]. These results showed no significant cytotoxic effect of CS-PLGA particles loaded with EO components < 5 µg/mL, supporting the results of MTT assay and cell proliferation analysis. Another important aspect to highlight is the cellular localization and uptake of particles which reflects their efficacy at the cell level and is influenced by their size, polydispersity index, coating polymers, and surface charge [53]. Hence, FITC-labeled chitosan was used to functionalize the surface of PLGA particles to be easily traceable, providing a feasible method to study the behavior and fate of the polymeric particles [54]. The most clinically relevant finding to emerge from the confocal image analysis was that the particles had a good cellular adhesive effect due to the cationic nature of the particles that induces electrostatic interaction with the anionic cell surface [27]. Additionally, the particles were found as intercellular, showing the ability of chitosan to enhance the permeability via opening the tight junctions of intestinal cells as explained in previous studies [20,55]. The cellular uptake of the particles was size-dependent, where the micro particulates were intercellularly localized and the nanoscale particles were taken up by the FKD-1-R cells and observed at a perinuclear localization, in agreement with previous studies [27,56].

The inhibition of parasite motility is considered the gold standard for evaluating the anthelmintic activity of components of EOs either in non-encapsulated (free) [57] or encapsulated form [58]. In the present study, we found that free, combined EO components (linalool, geraniol, and eugenol) have a strong effect on larval motility, reaching 96.1% inhibition, compared with EO components encapsulated in uncoated and CS-PLGA particles (75% and 76.9%, respectively), which may be related to the slow release of EO components from the PLGA particles. The synergistic effect of this combination compromises the integrity of the parasite cuticle and increases the permeability of EO components through the phospholipid layers of the parasites, leading to disruption of intracellular protein and lipids [59]. An additive effect of chitosan was observed as indicated by the reduced IC_50_ values in the CS-PLGA particles compared with the uncoated particles. This is related to the interaction of the positively charged chitosan with the negatively charged outer phospholipid layer of the parasite cuticle, which enhances the disruption of the channel system and reduces the metalloprotein activity via chelating with parasite cytoplasmic metals [42,60,61]. These results are further supported by scanning electron microscopy of larval and adult worm stages treated with the CS-PLGA particles, showing that adsorption of particles to the cuticle was associated with considerable structural damage, particularly with the nanoscale particles due to their ability to penetrate and permeate the cuticle. A similar observation was reported in a previous study [57], attributing this effect to the affinity of the hydrophobic EO components to the cell membrane, which alters the electrostatic balance and increases the cuticle’s permeability and damage of cuticular protein.

## 5. Conclusions

We encapsulated three EO components (linalool, geraniol, and eugenol) in CS-PLGA particles using a microfluidic technique with a high entrapping efficacy and loading capacity. These particles were characterized by micro- and nanoscopic sizes and monodispersed spherical shape, and a positive zeta potential after surface charge changing by chitosan coating. Solid-state characterization using FTIR, TGA, and XRD verified the encapsulation process and enhancement of thermal stability. The in vitro release studies revealed pH responsiveness of EO components from CS-PLGA particles in addition to a more sustained release than the uncoated PLGA particles. The encapsulation of EO components within CS-PLGA particles reduced the cytotoxic effect of EO components and did not have any significant adverse impact on the cell growth, viability, and cell membrane integrity compared with free EO components or uncoated particles. The particles were localized on the cell surface and intercellularly in case of large particles, whereas the nano-scale particles were found intracellularly. The CS-PLGA particles loaded with combined EO components exhibited a significant anthelmintic activity against the juvenile and adult stages of *H. contortus* and *T. axei* worms. The treatment efficacy was augmented by the synergistic effect of combining EO components and the additive effect of chitosan, which collectively caused significant ultrastructural damage of the parasite cuticle. These findings present CS-PLGA particles as a potential carrier for oral and post-ruminal delivery of encapsulated EO components to combat gut nematodes of sheep.

## Data Availability

Not applicable.

## Supplementary Materials

The following supporting information can be downloaded at: https://www.mdpi.com/article/10.3390/pharmaceutics14102030/s1, Figure S1: Representative gas chromatograms of crude oils of citronella, clove, coriander, and lavenders showing the retention times of the major constituents; Figure S2: The chemical structure of geraniol, linalool, and eugenol used in the study; Figure S3: GC/MS calibration curves used for quantification of three essential oil (EO) components (geraniol, linalool and eugenol) in crude EOs, working solutions and encapsulated particles; Figure S4: Calibration curves for UV-vis spectrophotometric determination of the release kinetics of essential oil components from uncoated and coated encapsulated PLGA particles; Figure S5: Optimization of cell seeding density. FKD-1-R cells were seeded at different densities 0.5, 1, 2, 3, 4, 5 and 6 × 10^4^ cells/well of 96-well tissue culture microtiter plates. After 24, 48 and 72 h, cell viability was measured by the MTT assay. A seeding density 2 × 10^4^ cells/well was chosen because higher seeding densities did not result in any discernible cell growth at 48 and 72 h; Table S1: Optimization of PLGA particle formulations based on average size and polydispersity index at different conditions including EO components concentration, polymer weight and solvent used (acetone or DCM or DCM and acetone at a ratio of 1:10 and 1:20); Table S2: The half maximal inhibitory concentrations (IC_50_) of the essential oil (EO) (µg/mL) in loaded PLGA particles, chitosan-coated PLGA particles and free EO components.
