# Supplementary material for: Microfluidic-Based Formulation of Essential Oils-Loaded Chitosan Coated PLGA Particles Enhances Their Bioavailability and Nematocidal Activity
Source: Pharmaceutics. 2022 Sep 23;14(10):2030. doi: 10.3390/pharmaceutics14102030 (PMC9608619; doi:10.3390/pharmaceutics14102030)
Supplement: Supplementary file 1 [file pharmaceutics-14-02030-s001.zip › pharmaceutics-1892042-supplementary.pdf]

Supplementary Materials:

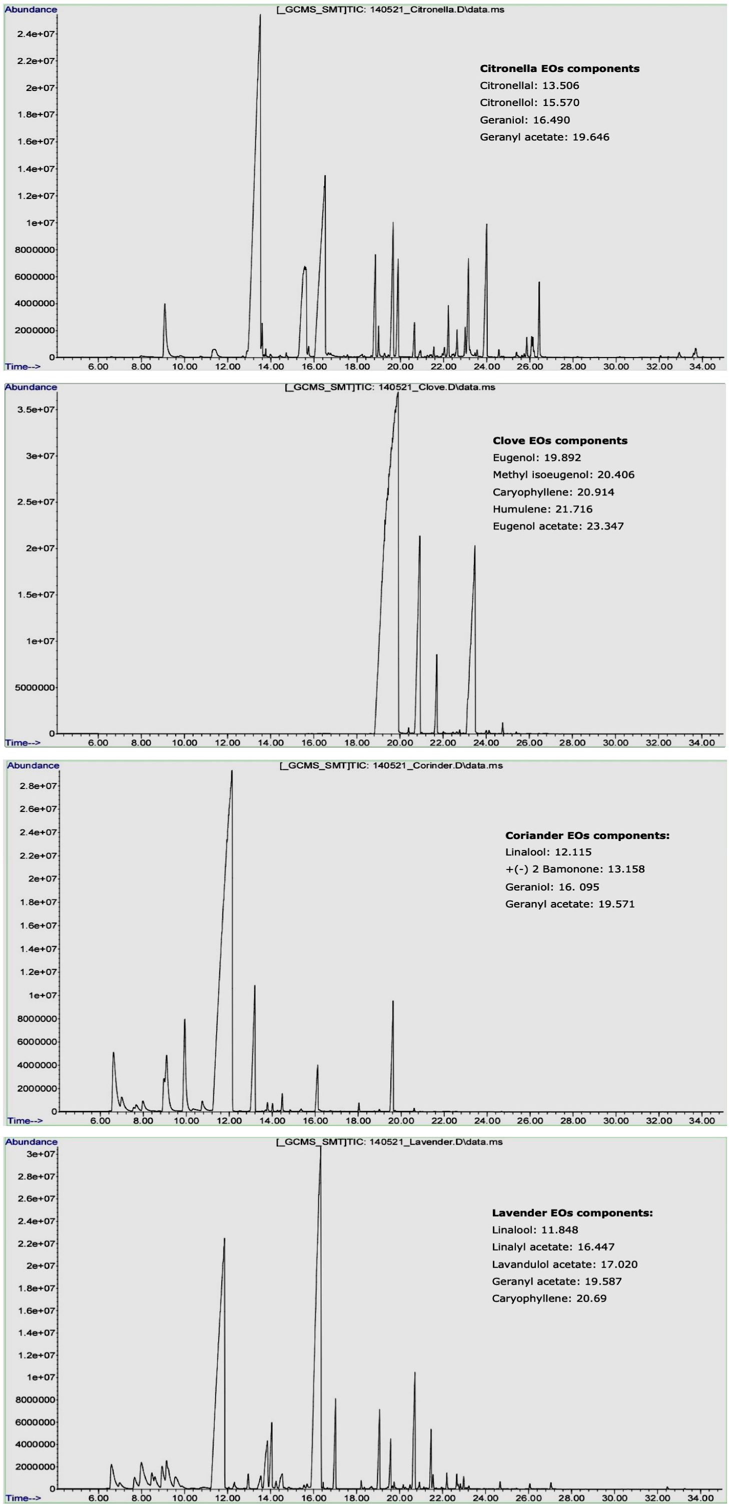

Figure S1. Representative gas chromatograms of crude oils of citronella, clove, coriander, and lavenders showing the retention times of the major constituents.

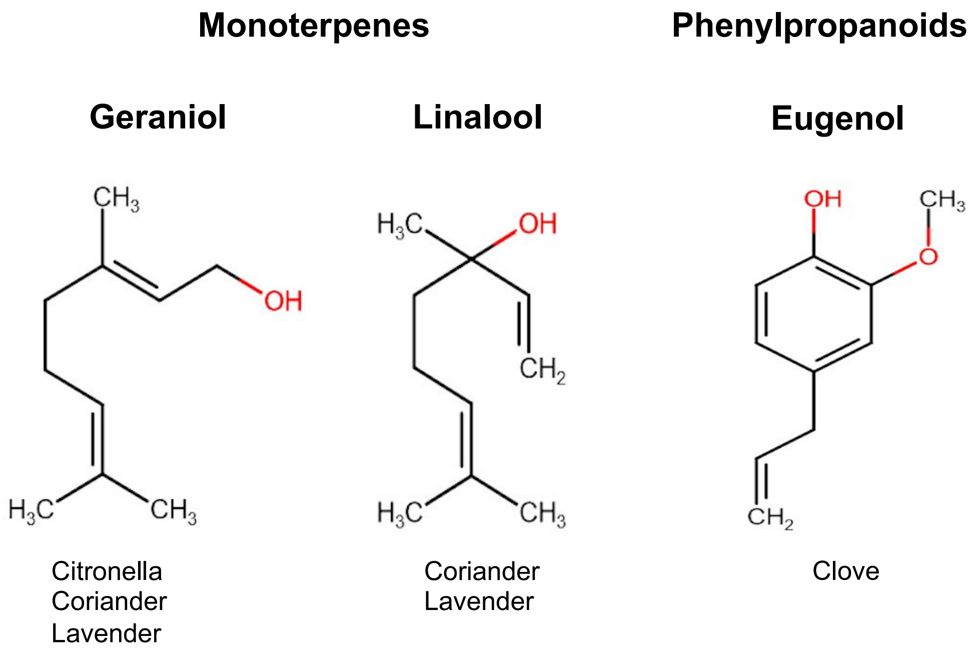

Figure S2. The chemical structure of geraniol, linalool, and eugenol used in the study.

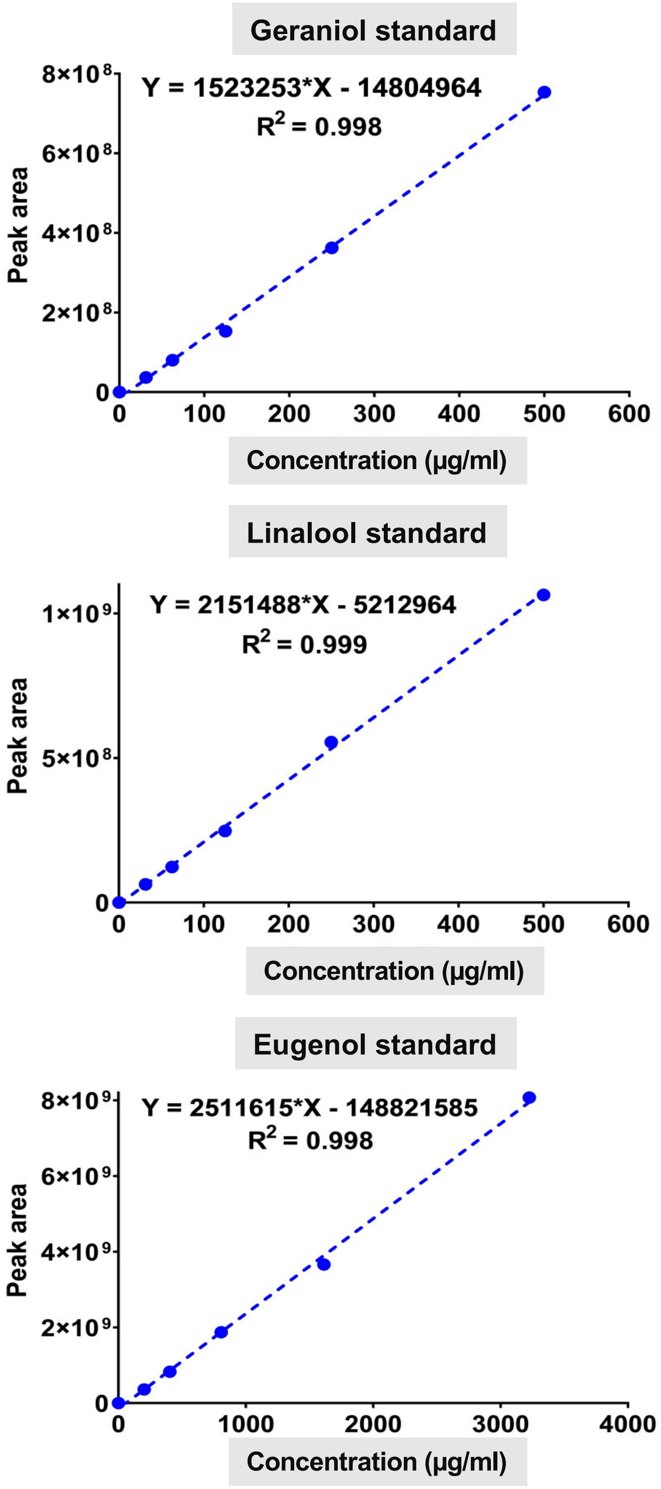

Figure S3. GC/MS calibration curves used for quantification of three essential oil (EO) components (geraniol, linalool and eugenol) in crude EOs, working solutions and encapsulated particles.

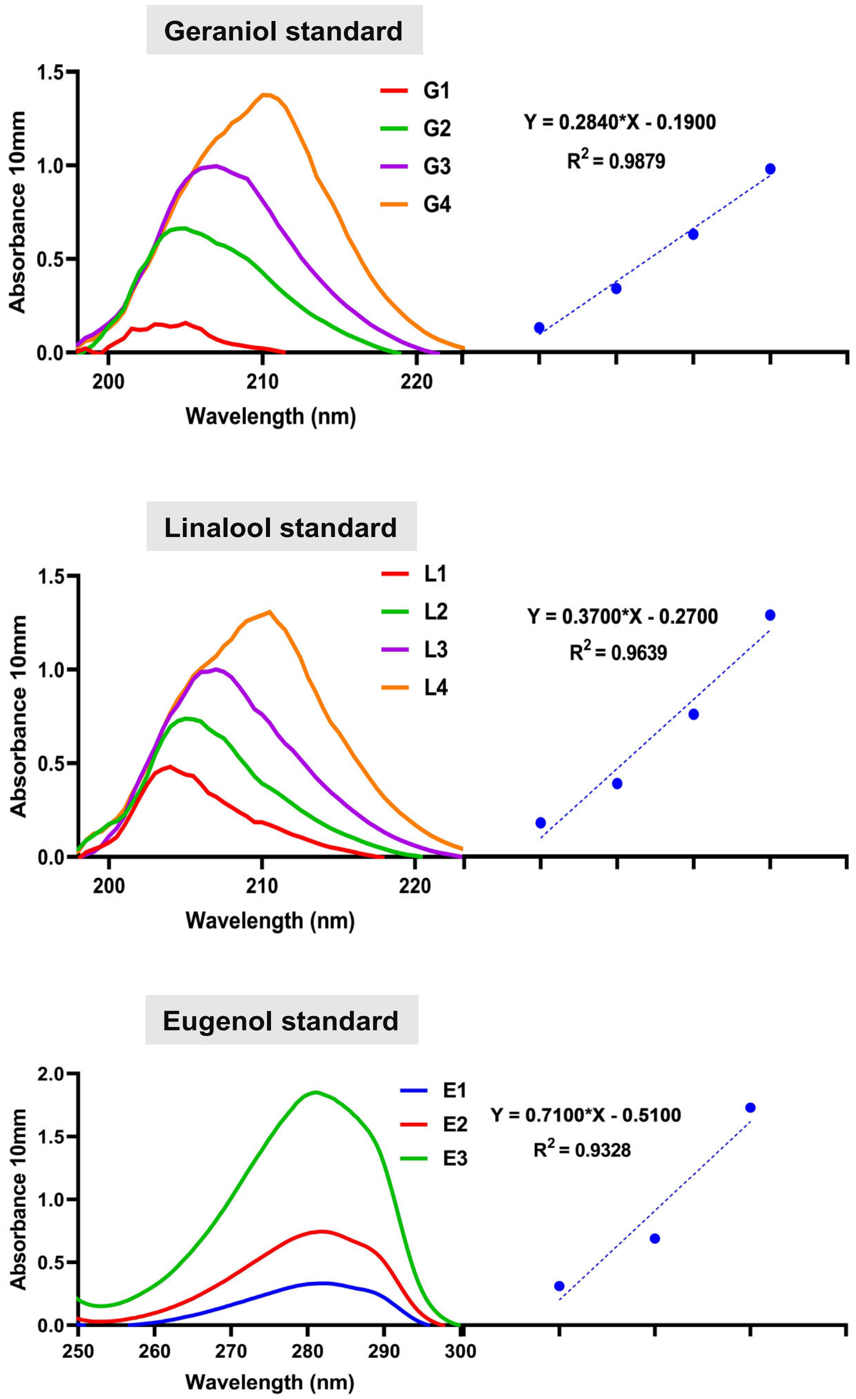

Figure S4. Calibration curves for UV-vis spectrophotometric determination of the release kinetics of essential oil components from uncoated and coated encapsulated PLGA particles.

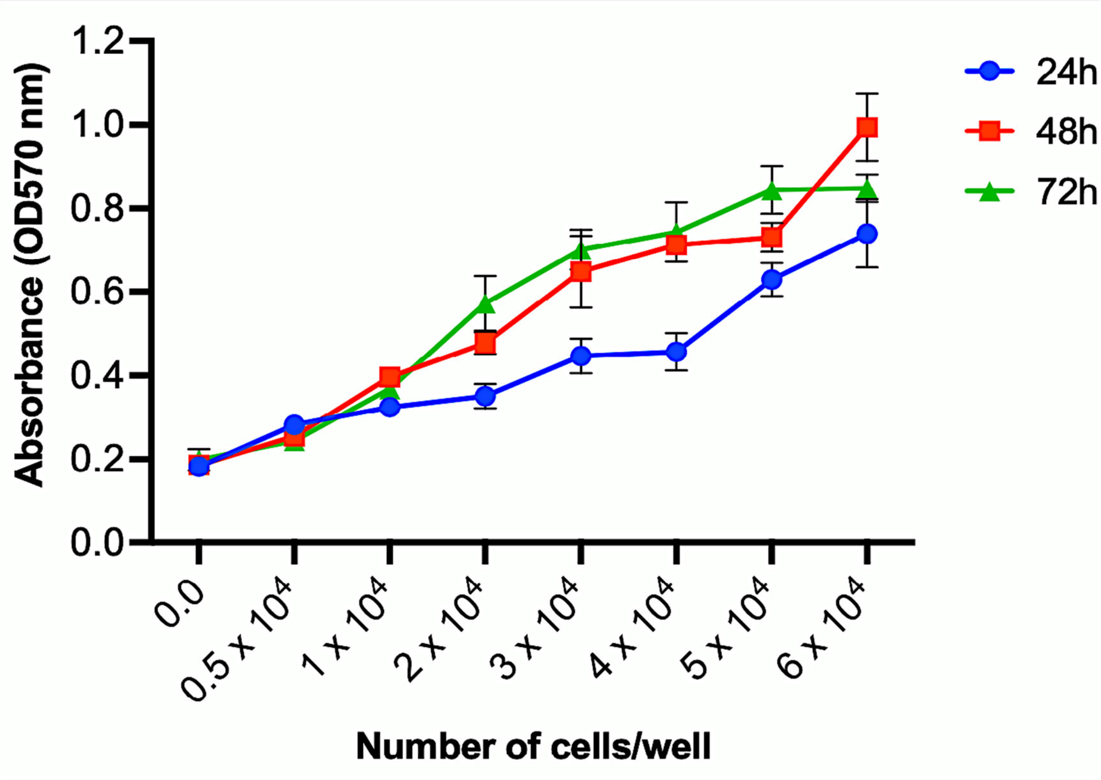

Figure S5. Optimization of cell seeding density. FKD-1-R cells were seeded at different densities 0.5, 1, 2, 3, 4, 5 and 6 x10^4 cells/well of 96-well tissue culture microtiter plates. After 24, 48 and 72 h, cell viability was measured by the MTT assay. A seeding density 2 x10^4 cells/well was chosen because higher seeding densities did not result in any discernible cell growth at 48 and 72 h.

Table S1. Optimization of PLGA particle formulation based on the average size and polydispersity index (PDI) at different conditions including essential oil (EO) components' concentration (%), polymer PLGA concentration (%) and the solvent used (acetone or DCM, or DCM and acetone at a ratio of 1:10 and 1:20).

Formulation	EO concentration (%)	PLGA concentration (%)	Solvent used	Average size (nm)	PDI
F1	0.25	1	Acetone	131.5 ± 18.2	0.23
F2	0.25	2	Acetone	189.8 ± 51.1	0.30
F3	0.50	1	Acetone	133.6 ± 98.7	0.32
F4	0.50	2	Acetone	192.9 ± 133.6	0.24
F5	1.00	1	Acetone	158.3 ± 9.8	0.20
F6	1.00	2	Acetone	211.3 ± 70.9	0.32
F7	0.25	1	DCM	448 ± 205.0	0.26
F8	0.25	2	DCM	525.6 ± 170.6	0.34
F9	0.50	1	DCM	537.5 ± 98.7	0.42
F10	0.50	2	DCM	554.8 ± 219.7	0.34
F11	1.00	1	DCM	742.2 ± 127.4	0.63
F12	1.00	2	DCM	751.1 ± 66.3	0.45
F13	0.50	1	1:10	296.3 ± 111.7	0.23
F14	0.50	2	1:10	301.5 ± 151.4	0.23
F15	0.50	1	1:20	313.6 ± 201.3	0.25
F16	0.50	2	1:20	361.0 ± 134.1	0.23

Table S2. The half maximal inhibitory concentrations (IC_{50}) of the essential oil (EO) (μg/mL) in loaded PLGA particles, chitosan-coated PLGA particles and free EO components.

Particle/EO	Geraniol	Linalool	Eugenol	Combined
PLGA	7.47 ± 0.43	3.92 ± 0.51	2.78 ± 0.55	1.47 ± 0.61
CS-PLGA	3.91 ± 0.51	0.91 ± 0.56	1.48 ± 0.49	0.74 ± 0.62
Free EOs	1.65 ± 0.79	0.67 ± 0.44	1.07 ± 0.83	0.58 ± 0.31
